# Incidence and clinical patterns of cutaneous leishmaniasis at a Tertiary hospital in Hadiya Zone, Ethiopia: a hospital-based cross-sectional study

**DOI:** 10.1186/s12879-026-13538-6

**Published:** 2026-05-12

**Authors:** Seblewengel Maru Wubalem, Helina Melis Kefetew, Saada Yasin Abduselam, Mihret Gezahegn Brussow, Shemsu Abraham Hussien

**Affiliations:** 1 Department of Pathology, Wachemo University, Hossana, Ethiopia; 2 Department of Dermatology, Wachemo University, Hossana, Ethiopia

**Keywords:** Cutaneous leishmaniasis, FNAC, Hadiya, Ethiopia

## Abstract

**Introduction:**

: Leishmaniasis, classified as a neglected tropical disease (NTD), poses significant health challenges in impoverished communities and has shown a rising incidence in recent years. This study examines the incidence of cutaneous leishmaniasis (CL) in the Nigist Eleni Mohammed Memorial Specialized Comprehensive Hospital (NEMMSCH) in the Hadiya Zone, Ethiopia. It represents the first study of CL conducted at this institution.

**Methods:**

This hospital-based cross-sectional study was conducted at NEMMSCH, the primary referral center for the Hadiya Zone. The methodology involved a retrospective review of medical and pathology records for dermatology patients diagnosed between September 2021 and February 2025.

**Results:**

Out of 105 clinically suspected cases, 60 were confirmed as CL through fine needle aspiration cytology. The incidence rate was found to be 2.7 per 1,000 dermatology patients, with a mean age of 23.95 years. Notably, both males and females were equally affected, while the majority of cases (71.7%) occurred in individuals over 15 years old. The study identified plaques as the most common clinical presentation, and lesions were predominantly localized to the face and upper extremities.

**Conclusion:**

The findings underscore the incidence of CL within the study area. Enhanced training for healthcare providers and the implementation of community-level preventive measures are critical to halting its expansion. Furthermore, these results have significant implications for surveillance and public health planning. This study advocates for the provision of improved diagnostic and treatment options, as well as further research to identify the specific risk factors contributing to the disease’s occurrence in the region.

**Supplementary Information:**

The online version contains supplementary material available at 10.1186/s12879-026-13538-6.

## Introduction

Leishmaniasis is categorized as a neglected tropical disease (NTD). NTDs comprise a group of long-lasting infectious diseases, primarily caused by parasites, which are highly prevalent in developing nations. These diseases often lead to significant illness but typically result in low mortality rates [1, 2]. Leishmaniasis is caused by protozoan parasites from over 20 species of Leishmania [3]. This flagellated protozoan belongs to the order Kinetoplastida and the family Trypanosomatidae [3]. More than 90 species of sand flies are responsible for transmitting Leishmania parasites [4].

The disease is most prevalent among impoverished communities in the world’s poorest nations [3, 5]. Leishmaniasis has shown a rising incidence in recent years. Factors associated with this increase include environmental changes such as climate change, deforestation, and urbanization, along with globalization and migration, which greatly influence the distribution of sand fly vectors and reservoir hosts. Furthermore, poverty and limited access to healthcare exacerbate the impact of leishmaniasis on vulnerable communities [6, 7]. Leishmaniasis is endemic in Ethiopia, where both CL and VL are growing health concerns [8, 9].

There are three primary forms of leishmaniasis, determined by the species of Leishmania involved: visceral leishmaniasis (VL), cutaneous leishmaniasis (CL), and mucocutaneous leishmaniasis (MCL) [3, 10, 11].

CL is endemic in over 70 countries globally, with 90% of cases occurring in Afghanistan, Algeria, Brazil, Pakistan, Peru, Saudi Arabia, and Syria [5, 10, 12]. CL can present in various forms, primarily as localized CL (LCL), mucocutaneous leishmaniasis (MCL), and diffuse CL (DCL). The infection typically begins as a small erythema that develops into a papule and nodule, which can ulcerate within 2 weeks to 6 months [5]. In healthy individuals, CL usually heals within 1 to 2 years if left untreated, due to the development of species-specific immunity; however, healing often results in disfiguring scars. MCL affects the mucosa and underlying connective tissue, while DCL is marked by non-ulcerative nodules that can spread and potentially cover the entire body of the patient [5, 10, 13]. Lesions persisting for more than three years are classified as chronic CL [14].

In Ethiopia, cutaneous leishmaniasis is mainly caused by *Leishmania aethiopica*, although there have been sporadic reports of *L. tropica* and *L. major* [15, 16]. There is also a case report of CL attributed to *L. donovani* [17], with additional cases reported in Sri Lanka [18]. The rock hyrax serves as the reservoir host [9], while known vectors include *P.pedifer*,* P. longipes*, and *P. sergenti* [9, 15, 16].

Leishmaniasis can be diagnosed through various methods, including cytology smears, histopathology, culture, serology, and molecular studies [19]. Cytological and histopathological techniques identify amastigotes or leishman-Donovan (LD) bodies under a microscope as round or oval structures measuring 2–4 μm in diameter, characterized by distinct nuclei and kinetoplasts. Fine needle aspiration cytology (FNAC) is preferred over scraping smears, as it offers a better yield and greater patient comfort [10].

Although CL is not life-threatening, it can have significant psychosocial impacts due to disfiguring lesions and permanent scarring. Consequently, prioritizing prevention, control, and treatment is essential. While CL remains a major public health challenge in Ethiopia, localized epidemiological data for the Hadiya Zone are scarce. This study represents the first research on CL conducted at the Nigist Eleni Mohammed Memorial Specialized Comprehensive Hospital (NEMMSCH). It aims to determine the incidence of CL at this referral hospital and assess its clinical features and demographic characteristics.

Identifying new endemic foci is crucial for the Ethiopian CL control program as it facilitates the transition from passive case detection to active surveillance, ensuring that diagnostic and therapeutic resources are deployed to these areas before transmission becomes unmanageable.

## Materials and methods

### Study setting

The study was conducted in the Departments of Pathology and Dermatology at the College of Health Sciences, NEMMSCH, Wachemo University, located in the Hadiya Zone of the Central Ethiopia Region. NEMMSCH is the primary referral hospital in the Zone providing specialized pathology and dermatology services. It is located in Hossana, approximately 230 km from the nation’s capital. The pathology department examines an annual average of 719 biopsies, 1,807 FNAC samples, 474 hematology samples, and 174 body fluid cytology samples. The department is staffed by two pathologists, two general practitioners, four laboratory technologists, and four support staff. FNAC sampling, smear preparation and microscopic interpretation is performed by pathologists. Following sampling, air-dried smears are stained with Wright stain for microscopic examination.

### Study design and period

This research employed a hospital-based cross-sectional design involving a retrospective review of medical and pathology records. The study included dermatology patients who visited NEMMSCH between September 2021 and February 2025. Data collection was conducted from January 15 to February 28, 2025.

### Source and study population

The source population consisted of all dermatology patients evaluated at the hospital during the study period. The study population included all patients with clinically suspected cutaneous leishmaniasis (CL).

### Eligibility criteria

All patients with clinically suspected CL were included in the study. Cases where the diagnosis could not be validated through FNAC were excluded.

### Operational definition

**Fig. 1:**
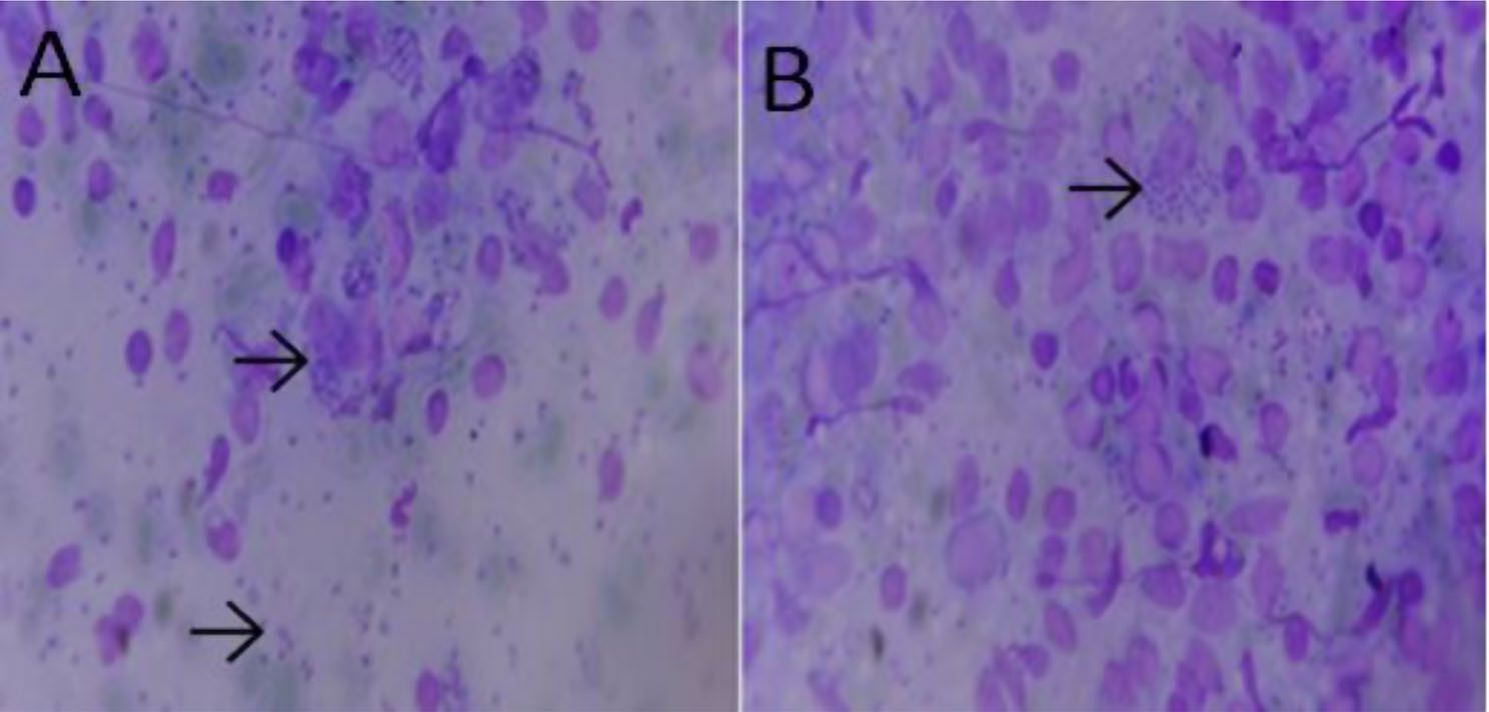
Microscopic images of FNAC smears from cutaneous leishmaniasis: smears showing intra- and extracellular LD bodies (arrow) and epithelioid macrophages (**A**: 20×; **B**: 40×; Wright stain)

In this study, a case of CL was defined as a diagnosis rendered by a senior anatomic pathologist following the microscopic examination of FNAC slides. FNAC was performed by pathologists within the department, and the air-dried smears were stained with Wright’s stain. The diagnosis of CL was confirmed if amastigotes (LD bodies) accompanied by chronic inflammation with or without granulomas were detected on the smears (Fig. 1).

### Data collection, quality control, and analysis

A structured data extraction tool was used for data collection. Demographic data, clinical presentations, and cytopathological findings were extracted from the patients’ medical records and pathology report archives. To ensure data quality, training was provided to the data collectors prior to the start of the study. A pre-test involving about 5% of the patients was conducted to evaluate the tool, leading to necessary adjustments. All questionnaires were reviewed for during and after the data collection phase. All questionnaires were assessed for completeness and accuracy both during and after the data collection phase. The questionnaire was created specifically for this study and has not been published elsewhere before. Details about the questionnaire can be found in Supplementary File 1.

Throughout this process, regular supervision was performed, with the supervisor daily verifying the data for completeness and accuracy.

The collected data were entered, cleaned, and analyzed using version 26 of the Statistical Package for the Social Sciences (SPSS) software. Descriptive statistics were used to summarize the data, and the Chi-Square Goodness-of-Fit test was employed to determine if the observed frequencies of clinical and demographic variables differed significantly from expected distributions.

## Results

There were a total of 105 clinically suspected cases of cutaneous leishmaniasis in the study period. Out of these cases 56 (53.3%) were males and 49 (46.7%) were females with a mean age of 23.95. Among these, 60 of the suspected cases confirmed to have cutaneous leishmaniasis by FNAC demonstrating LD bodies accompanied by chronic inflammation with or without granuloma (Fig. 1), therefore included in the study. The rest 45 case showed only granulomatous inflammation.

A total of 22,269 patients visited the hospital’s dermatology clinic during the study period, resulting in an incidence of 2.7 confirmed cases of CL per 1,000 dermatology patients. Among the 60 cytologically confirmed cases, 30 (50%) were male and 30 (50%) were female. Patient ages ranged from 1 to 65 years, with a mean of 23 years and a median of 20.5 years (Table 1). A Chi-Square Goodness-of-Fit test was conducted to compare the proportion of cases above and below the age of 15, revealing a significant difference: a significantly higher proportion of patients, 43 (71.7%), were above the age of 15, χ^2^ (1, *N* = 60) = 11.27, *p* = .001.

**Table 1:** Demographic characteristics of cutaneous leishmaniasis patients attending Nigist Eleni Mohammed Memorial Specialized Comprehensive Hospital between September 2021 and February 2025 (*n* = 60)

Variable	Category	Frequency	Percentage
Sex	Male	30	50
	Female	30	50
Age group in year	1–10	9	15
	11–20	21	35
	21–30 31–40 41–50 51–60 61–70	17 6 2 4 1	28.3 10 3.3 6.7 1.7
Districts	Lemo	22	36.7
	Analemo	21	35
	Hossana	8	13.4
	Gibe	3	5
	Soro	2	3.3
	Duna	1	1.7
	Durame	1	1.7
	Enemor	1	1.7
	Hadero	1	1.7

Analysis of the geographic distribution across the nine districts also showed a significant difference in the proportion of patients by location. The highest number of patients originated from Lemo (*n* = 22) and Analemo (*n* = 21), which together accounted for 71.7% of total cases, χ ^2^ (8, *N* = 60) = 90.90, *p* < .001. Specifically, the highest rates were observed in the Belesa sub-district (Lemo) and Fonko sub-district (Analemo), each recording 9 cases (15%). Additionally, eight cases (13.4%) were from Hossana, the zonal capital. Temporally, the highest proportions of cases occurred in June and August (9 cases, 15% each), while the lowest were recorded in March and October (2 cases, 3.3% each).

**Fig. 2:**
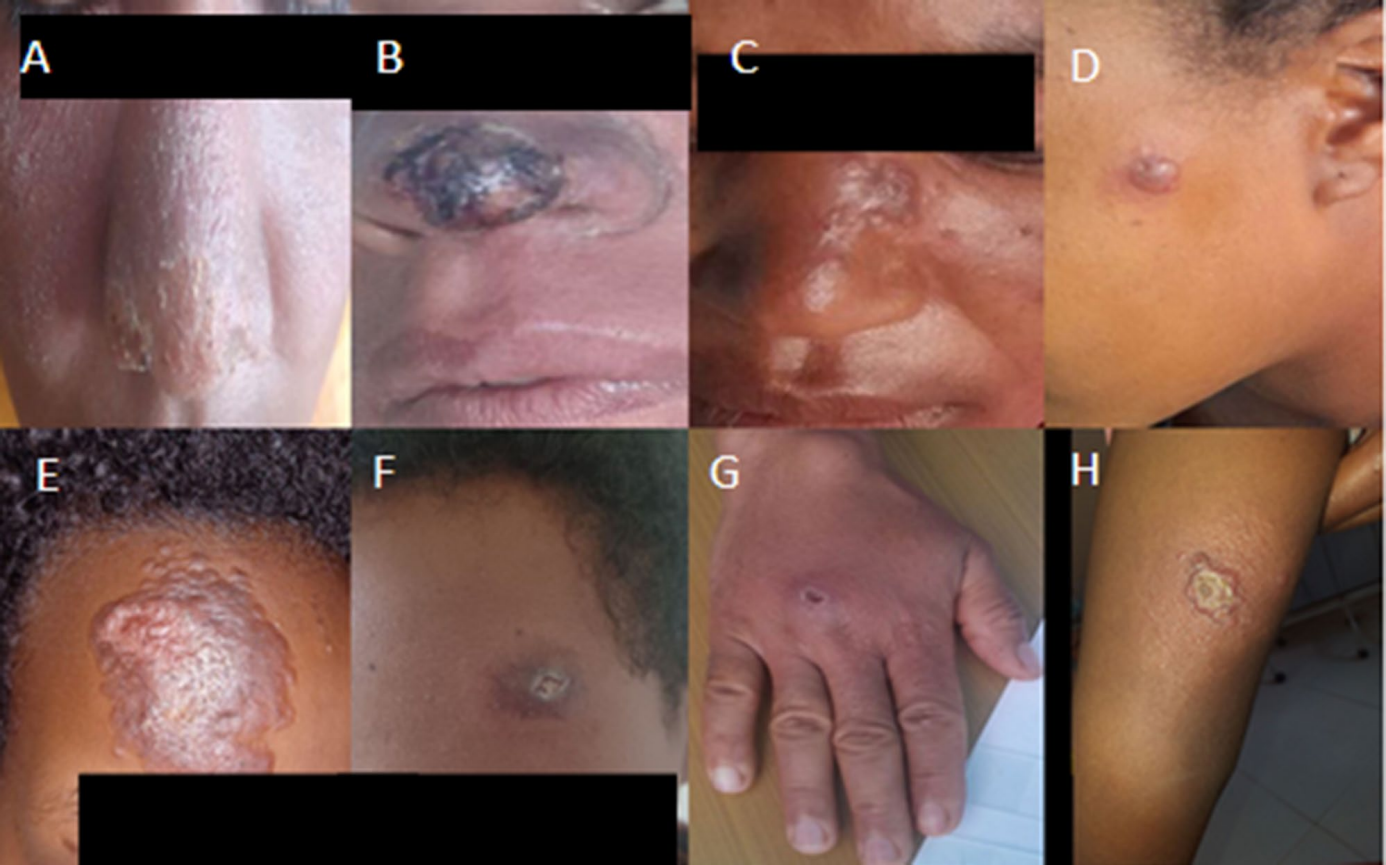
Clinical images of cutaneous leishmaniasis: (**A, B**) ulcers on the nose; (**C, D, E**) plaques involving the nose, cheek, and forehead, respectively; (**F, G, H**) ulcerated lesions on the forehead, dorsum of the hand, and forearm, respectively

Plaque is the most frequent clinical presentation, accounting for 46 (76.6%) cases. Seventeen (28.3%) of the cases have mucosal involvement. A significant proportion of patients (55, 96.5%) present later than one month from the onset of the lesion, most of them within the first six months. The face and upper extremities are frequently involved sites (Fig. 2). All of the lesions are below 5 cm. Only 8 of the cases had multiple lesions (Table 2).

**Table 2:** Clinical findings of cutaneous leishmaniasis patients attending Nigist Eleni Mohammed Memorial Specialized Comprehensive Hospital between September 2021 and February 2025 (*n* = 60)

Variable	Category	Frequency	Percentage
Type of leshmaniasis	Localized cutaneous	43	71.7
	Mucocutaneous	17	28.3
Type of lesion	Plaque	46	76.7
	Ulcer	14	23.3
Number of lesion	Single	52	86.7
	Multiple	8	13.3
Duration of lesion (*n* = 57)*	1–6 month	36	63
	7–12 month	19	33.7
	> 12 month	2	3.5
Site of lesion	Face	49	81.7
	Upper extremity	7	11.7
	Face and upper extremity	2	3.3
	Lower extremity	1	1.7
	Neck	1	1.7
Part of the face involved (*n* = 51)	Nose	18	30
	Cheek	13	21.7
	Forehead	6	10
	Lip	5	8.3
	Cheek and nose	4	6.7
	Nose and forehead	2	3.3
	Chin	3	5

* Duration of lesion was available for 57 of the 60 total cases; data was missing for 3 cases

## Discussion

The study revealed that the incidence of CL in the hospital was 2.7 per 1,000 dermatology patients. CL remains a significant public health concern, spreading to various regions across Ethiopia. Historical records of CL in Ethiopia date back to 1913. Notably, the Hadiya Zone was not previously recognized as endemic for leishmaniasis. However, recent observations have noted an increase in CL cases at the Zone’s referral hospital, particularly in the dermatology and pathology departments. A risk assessment map developed by Ahmed et al. categorizes this area as having a moderate to high risk for cutaneous leishmaniasis [15]. The closest documented outbreak occurred in the Silti Zone in 2008, located to the north of the Hadiya Zone [20]. Additionally, Ochollo village in the Gamo Gofa Zone, approximately 230 km south of the Hadiya district, is the next closest area known to be endemic for CL [21].

In this study, the highest incidence of CL was observed in the two districts situated in the northern part of the Hadiya Zone: Lemo and Analemo. These districts border the Silte Zone, where a CL outbreak has been reported previously [20]. Therefore, one potential contributing factor to the increased incidence in these areas could be the migration of sand fly vectors and reservoir hosts from neighboring regions. However, further research is needed to identify all the possible environmental risk factors associated with this surge.

The equal incidence of CL in male and female patients was observed in this study, matching findings from different studies in Ethiopia [21, 22]. In contrast, a higher incidence in males is seen in Gondar, Ethiopia, and Nepal [23, 24]. In this study, CL was most frequently observed in the age group of 11–20 years, accounting for 22 cases (36.7%), similar to trends seen in the Silti Zone [20]. This may be due to the fact that this age group is actively involved in outdoor activities, which increases the likelihood of being bitten by sandflies.

Unlike endemic regions where CL prevalence is high in those under the age of 15 and decreases thereafter due to the development of immunity [5, 22], its prevalence is higher in individuals over the age of 15, accounting for 71.7% of cases. This suggests a lack of community-wide immunity, likely because the region was not previously endemic for CL infection.

The study showed that 43(71.7%) cases had LCL and 17(28.3%) of cases had MCL. Out of 17 MCL cases, 12 involved the nose and 5 involved the lip. Other studies also found that the incidence of MCL is between 5% and 20% of CL cases, with the nose being the most commonly affected part of the body [25, 26]. MCL is associated with more serious complications than LCL such as destruction of the oronasopharyngeal mucosa, and cartilaginous facial and upper airway structures, leading to disfigurement, secondary infection, and airway obstruction [27]. All of the patients presented with either plaques or ulcers; the earliest features of CL, such as papules [27], were not observed in our patients, as most presented more than one month after the appearance of the lesion.

In most cases, the duration of the lesion at the time of hospital visit is six months or less, accounting for 31 cases (63%). In contrast, a study from Nepal indicated that only 31% of cases present to the hospital within this timeframe [23]. Additionally, two studies from Ocholo village in southern Ethiopia found that the duration of the lesion in most patients exceeds six months, with an average duration of one year [21, 28]. Given that this condition was not endemic in the area, individuals may be particularly concerned about the appearance of new disfiguring wounds on exposed areas and may be unaware of the potential consequences. This concern could lead them to seek medical attention earlier than those residing in endemic regions.

Exposed parts of the body, mainly the face followed by the upper extremities, are affected by CL, together accounting for 58 cases (96.6%). Similar findings have been observed in studies conducted in the Silte Zone, Tigray, and Somali regions of Ethiopia and Nepal [20, 22, 23, 29]. The face is the most accessible body part and the preferred site for feeding by sandflies due to its softness [20].

Most of the lesions are single, with 52 cases (86.7%) being less than 5 cm in size. This makes treating patients with local therapy feasible. Local therapy is the preferred method of treatment if the lesions are fewer than five and smaller than 5 cm. Local therapies include intralesional antimonials, cryotherapy, and thermotherapy [10, 30]. In our setup, most patients used to be referred to centers where CL treatments were available until the beginning of cryotherapy services a year ago.

### Limitations

This study utilized a hospital-based cross-sectional design, which entails certain limitations. Due to the retrospective nature of the data collection, some patient records may have been missing or incomplete. Furthermore, only cytologically confirmed cases were included owing to the lack of alternative diagnostic modalities at the hospital; this likely resulted in an underestimation of the true incidence of CL. Finally, as a hospital-based study, these findings may not fully reflect the exact community-level incidence of CL within the broader population.

## Conclusion

In conclusion, this study highlights the incidence of cutaneous leishmaniasis (CL) at NEMMSCH in the Hadiya Zone. Our findings indicate that both males and females are equally affected. The incidence of CL is higher in individuals over the age of 15, suggesting that the older population in the area does not develop immunity, in contrast to those living in endemic areas.

The researchers recommend the implementation of advanced diagnostic tests, such as biopsies and molecular tests, along with appropriate treatments to ensure timely diagnosis and management of patients, thereby reducing unnecessary referrals. Furthermore, we advocate for targeted training to raise awareness among healthcare professionals regarding the occurrence of CL to prevent clinical mismanagement. At the community level, it is crucial to prioritize awareness campaigns and vector control efforts to halt disease transmission. Finally, expanded community-based and multi-center studies are necessary to define the precise incidence and environmental risk factors in Hadiya Zone and neighboring regions. Early identification of new endemic foci is essential for the success of national CL control programs.

## Supplementary Information

Below is the link to the electronic supplementary material.

Supplementary Material 1

## Data Availability

No datasets were generated or analysed during the current study.

## Abbreviations

CL: Cutaneous Leishmaniasis
DCL: Diffuse Cutaneous Leishmaniasis
FNAC: Fine Needle Aspiration Cytology
LCL: Localized Cutaneous Leishmaniasis
LD bodies: Leishman-Donovan bodies
MCL: Mucocutaneous Leishmaniasis
NEMMSCH: Nigist Eleni Mohammed Memorial Specialized Comprehensive Hospital
NTD: Neglected Tropical Disease
SPSS: Statistical Package for the Social Sciences
VL: Visceral Leishmaniasis
