# Supplementary material for: Incidence and clinical patterns of cutaneous leishmaniasis at a Tertiary hospital in Hadiya Zone, Ethiopia: a hospital-based cross-sectional study
Source: BMC Infect Dis. 2026 May 12;26:1260. doi: 10.1186/s12879-026-13538-6 (PMC13343574; doi:10.1186/s12879-026-13538-6)
Supplement: Supplementary file 1 — Supplementary Material 1 [file 12879_2026_13538_MOESM1_ESM.pdf]

Cutaneous Leshmaniasis Data Extraction Form

serial number

____

year at first presentation

____

month at first presentation

- ○ jan
- ○ feb
- ○ mar
- ○ apr
- ○ may
- ○ june
- ○ july
- ○ aug
- ○ sep
- ○ oct
- ○ nov
- ○ dec

woreda

____

kebele

____

Age (in year)

____

Sex

- ○ Male
- ○ Female

type of leshmaniasis

- [ ] localized cutaneous leshmaniasis
- [ ] mucocutaneous leshmanisis

type of lesion

- ○ plaque
- ○ ulcer

duration of lesion (in month)

____

size of lesion (in centimeters)

____

site of lesion

- [ ] face
- [ ] upper extremity
- [ ] lower extremity
- [ ] thrunk
- [ ] neck

if face which part of face

- [ ] cheeck
- [ ] nose
- [ ] forehead
- [ ] chin
- [ ] lip
- [ ] ear

number of lesion

- ○ single
- ○ multiple

if the lesions are multiple, how many?

____

FNAC finding

- [ ] granuloma
- [ ] granuloma and LD bodies
